# Ludwig's Angina: The Original Angina

**DOI:** 10.1155/2013/974269

**Published:** 2013-05-22

**Authors:** Karim Kassam, Ashraf Messiha, Manolis Heliotis

**Affiliations:** Division of Maxillofacial Surgery, Northwick Park Hospital, Northwick Park, London HA1 3UJ, UK

## Abstract

Ludwig's angina was first detailed by the German surgeon Wilhelm Friedrich von Ludwig in 1836. We present a case which needed awake fibreoptic intubation due to severe trismus and a prolonged period intubated in the Intensive Care Unit after incision and drainage of neck spaces and removal of his lower wisdom teeth. He was finally discharged a week after admission and followed up in the outpatient clinic. The case is presented with clinical photographs and a video of the fibreoptic intubation to illustrate the airway.

## 1. Background

The case is important as it illustrates the need to recognize that the early treatment of disease is necessary to avoid disastrous consequences and the importance to liaise with anaesthetic colleagues in order to keep the patient intubated for a period of time in order for the postoperative swelling and oedema to settle.

## 2. Case Presentation

A 25-year-old presented with a 3-day history of progressive difficulty of swallowing, odynophagia, dysphonia, trismus, extraoral swelling, and pain. On further questioning, the pain initiated in the right posterior mandible and progressed to the contralateral side.

He was immediately commenced on broad spectrum antibiotics with fluid resuscitation. He was also given regular dexamethasone and adrenaline nebulisers as needed if there were any episodes of respiratory distress.

On examination, he was febrile, normocardic with bilateral submandibular swelling, and raised floor of mouth. The interincisal distance was only 10 mm restricted by the swelling and pain. There was frank pus discharging from the operculum of the partially erupted lower right wisdom tooth.

**Figure 1:**
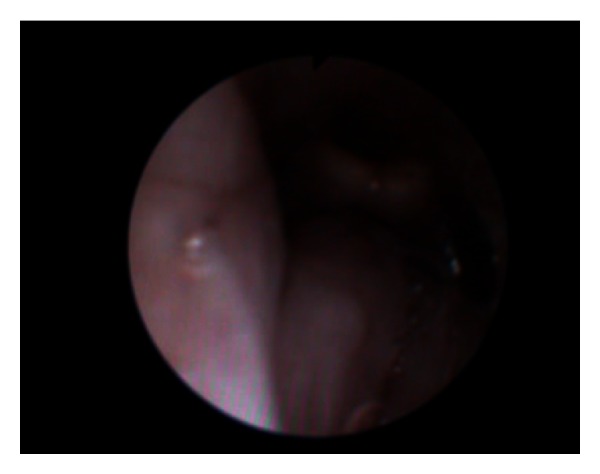
Video of the fibreoptic intubation illustrating swollen base of tongue and swollen epiglottis.

He was kept in the Accident and Emergency Resuscitation for one-to-one monitoring until he was taken to the operating theatre for intubation (Figure 1). 

Intraoperatively, the wisdom teeth were removed bilaterally along with bilateral decompression of the submandibular, sublingual, and submental spaces and the right buccal, lingual, submasseteric, and pterygomandibular spaces (Figures 3 and 4).

Copious amounts of pus came from the tooth socket of the lower right wisdom tooth and submandibular spaces.

Corrugated drains were sutured in placed for 5 days.

**Figure 2:**
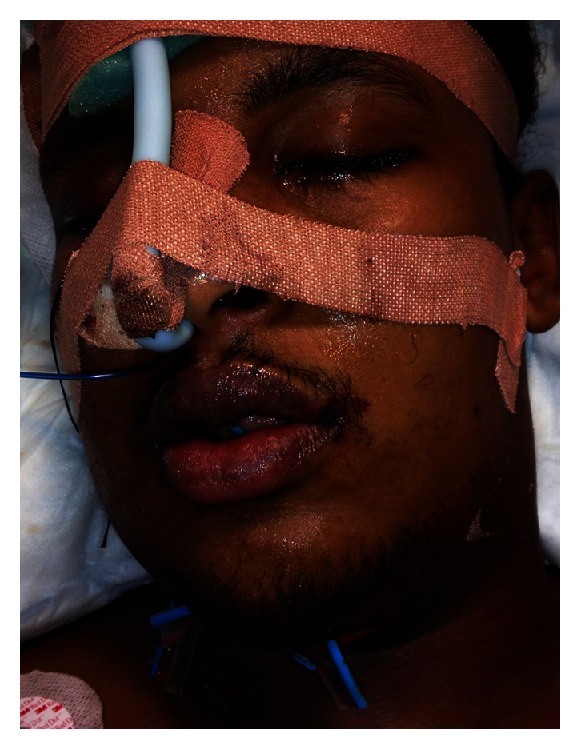
Patient intubated with drains in the Intensive Care Unit.

**Figure 3:**
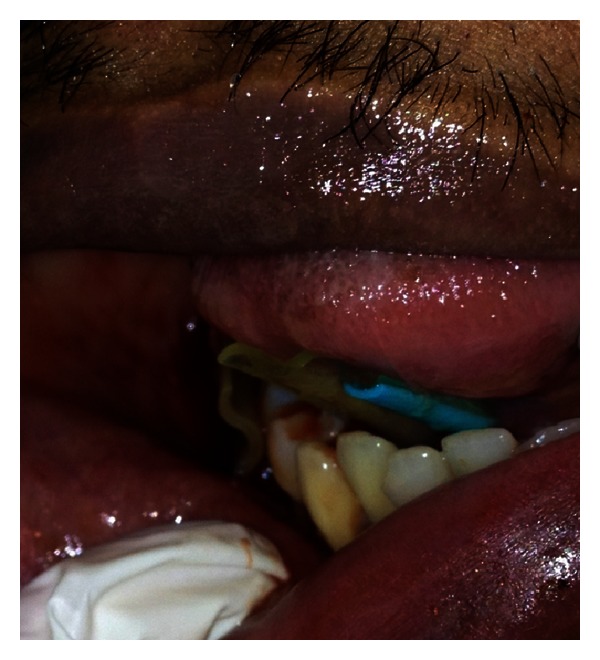
Closeup view of drain in the floor of the mouth “through and through” from submandibular space through the mylohyoid muscle (right drain in picture) and one in submasseteric space (left drain).

**Figure 4:**
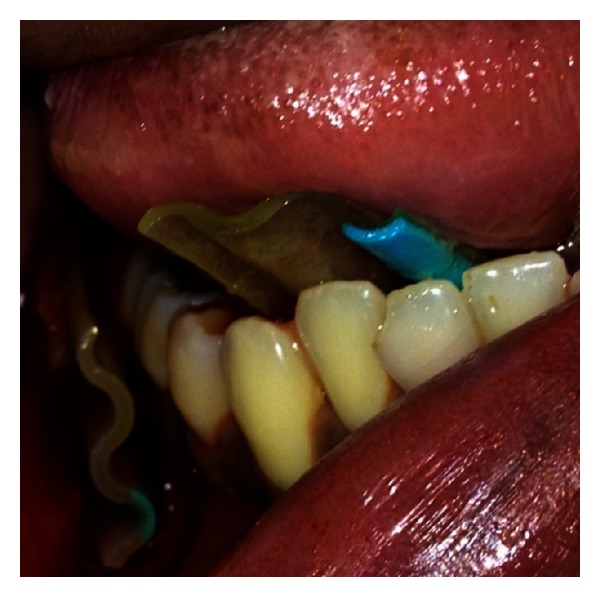
Closeup view of intraoral drains.

**Figure 5:**
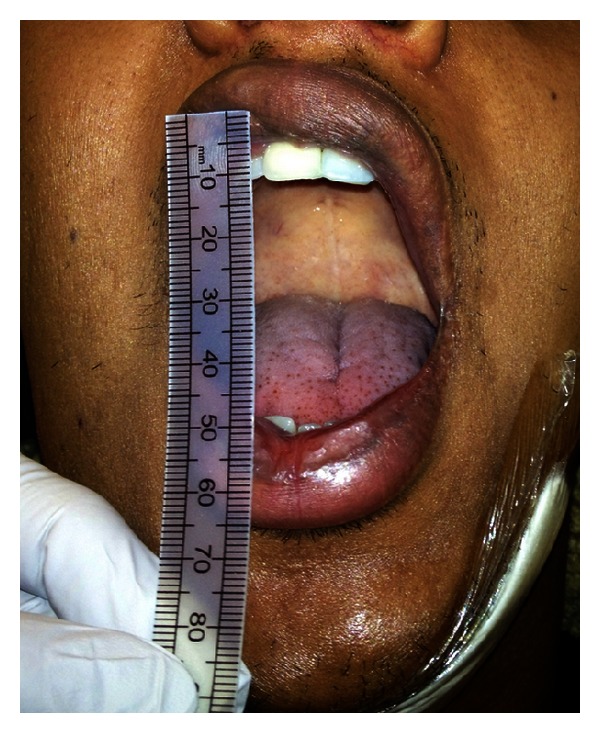
2-week postoperatively. Note the 40 mm interincisal distance and unrestricted mouth opening.

The patient was kept intubated for 72 hours (Figure 2) before being safely extubated and transferred to the ward. All the drains were removed by the 6th postoperative day, and the patient was discharged on the 7^th^ (Figure 5).

## 3. Discussion

**Figure 6:**
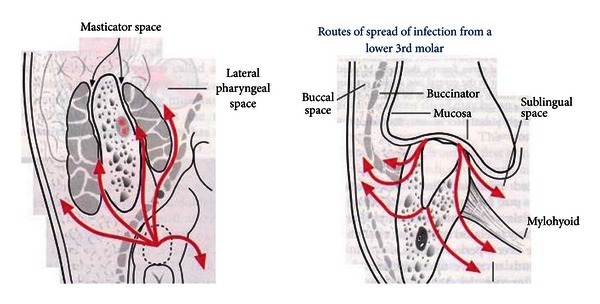
Routes of spread from a lower wisdom tooth. (Taken from http://www.exodontia.info).

**Figure 7:**
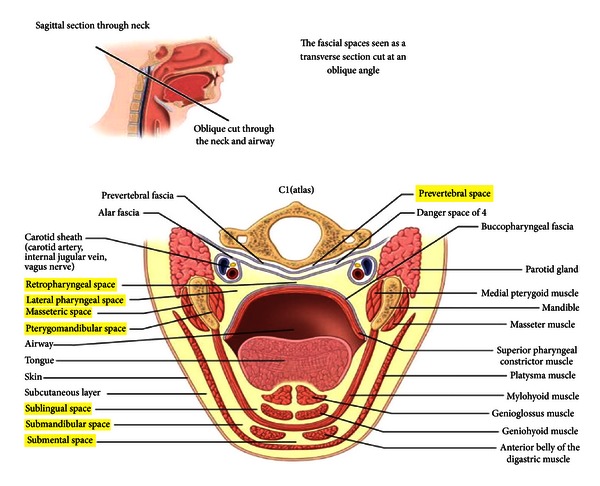
Close proximity of the posterior mandible to the prevertebral spaces which can lead directly to the mediastinum. (Taken from http://www.exodontia.info).

Ludwig's angina is a rapidly progressive, potentially fulminant cellulitis involving the sublingual, submental, submandibular and parapharyngeal spaces (Figures 6 and 7). The commonest cause is an infected lower wisdom, and this is seen in our practice.

Ludwig begins as a mild infection and progresses to induration of the upper neck with pain, trismus and tongue elevation. The most serious complication of course is respiratory embarrassment. It is therefore essential to act quickly so as not to lose the airway.

Angina is derived from the Latin word *angere* which means to strangle. Ludwig's angina appropriately describes deep neck abscesses in which the swelling of critical spaces which threaten to elevate the floor of the mouth displaces the tongue posteriorly and thereby strangles the patient.

In our case, our patient had severe trismus which is suggestive of involvement of the submasseteric space which was explored thoroughly.

Ludwig's angina preantibiotic era carried a very high mortality rate of around 50%, but it is still considerably high today at around 8%–10% [1]. The bacterial agents commonly isolated include streptococci viridans, staphylococcus aureus and staphylococcus epidermidis. Only 7% of Ludwig's angina cases are due to group A b-haemolytic streptococcus [2]. Early antibiotic treatment should be broad spectrum to cover gram-positive and gram-negative bacteria as well as anaerobes. Penicillin, metronidazole, clindamycin, and ciprofloxacin are often the antibiotics of choice. 

Blind nasal intubation should be avoided as it could cause bleeding, laryngospasm, oedema of the airway, rupture of pus into the oral cavity, and aspiration. 

Although distorted anatomy, oedema, and secretions may contribute to difficulty with fibreoptic intubation, in skilled hand, flexible fibreoptic nasal intubation is the preferred method of airway management [3] with high rates of success [4]. Elective awake tracheostomy is performed in our unit if fibreoptic intubation is not possible and of course cricothyroidotomy or emergency tracheostomy if the need arises. This echoes the “Practice Guidelines for the Management of the Difficult Airway” that were adopted by the American Society of Anaesthesiologists in 1992 and updated in 1993 [4]. Recently, the trend in terms of management of Ludwig's angina and deep neck infections has evolved from aggressive airway management into a more conservative one [5]. Wolfe et al. [5] conducted a retrospective analysis of all deep neck abscesses treated within a seven-year period. A total of 65% of their patients had airway compromise. Moreover, 42% of these patients required advanced airway control techniques. In this particular series, no surgical airway was required for the patients. In contrast, Mathew et al. [6] conducted a five-year retrospective study of their patients presenting with odontogenic maxillofacial space infections. A total of 14.6% of their patients presented with Ludwig's angina, and their preferred method for maintaining the airway was tracheotomy to endotracheal intubation. Potter et al. [7] compared tracheotomy versus endotracheal intubation for airway management in deep neck infections. They concluded that the use of tracheotomy permitted earlier movement to a noncritical unit and was associated with fewer intensive care costs and less overall cost of hospitalisation.

In our unit, it is common practice when patients have bilateral neck swellings and trismus to keep the patient intubated for 24–48 hours if they have been orally intubated or fibre-optic nasal intubation. This is to allow the oedema to settle which will inevitably get worse postoperatively and can compromise the airway further. In the past, there have been occasions in our unit where the patient has been extubated postoperatively and needed to go back to theatre for an emergency awake tracheostomy, hence, the prolonged intubation. Each case should obviously be taken at its own merit, and these are by no means stringent guidelines.

## 4. Conclusion

Ludwig's angina is potentially a life threatening condition and should be treated with respect. Broad spectrum antibiotics, surgical drainage, and airway management are paramount to prevent respiratory failure.

*Learning Points*

- Recognize the condition early.
- Commence broad spectrum antibiotics and steroids.
- Do not waste time imaging patient.
- Early communication with anaesthetist.
- Awake fibre-optic nasal intubation with surgeon on stand by for surgical airway.
- If nasal intubation is successful, consider prolonged intubation to avoid tracheostomy which has its own set of complications.

## Supplementary Material

Video of fibre-optic nasal intubation illustrating swollen base of tongue and epiglottis.
